# Influence of Multiple Used Implant Drills on Their Cutting Performance and Fracture Resistance

**DOI:** 10.3390/ma16155271

**Published:** 2023-07-27

**Authors:** Vasilios Alevizakos, Richard Mosch, Constantin von See

**Affiliations:** Research Center for Digital Technologies in Dentistry and CAD/CAM, Danube Private University, Steiner Landstrasse 124, 3500 Krems an der Donau, Austria; richard.mosch@dp-uni.ac.at (R.M.); constantin.see@dp-uni.ac.at (C.v.S.)

**Keywords:** zirconia implant drills, cutting performance, bending strength, drill usage, dental implants

## Abstract

This study aimed to analyze the influence of multiple uses of zirconia implant drills on their cutting performance and bending strength. The hypothesis was that drill usage and sterilization cycles would not affect drilling time or flexural strength. Sixty zirconia twist drills from Z-Systems were used to drill in the angulus mandibulae region of fresh porcine jaws. The drills were divided into four groups based on the cycle count, and the drilling time was measured. Bending strength tests were conducted using a universal testing machine, and statistical analysis was performed using ANOVA tests. The results showed that drilling times followed a normal distribution, and significant differences were observed in drilling times between group 1 and the other groups for the pilot drill. However, no significant differences were found for ø3.75 mm and ø4.25 mm drills, and drilling times also varied significantly among different drill diameters, regardless of the cycle count. Flexural strength did not significantly differ among drill diameters or sterilization cycles. Overall, using and sterilizing zirconia implant drills had no significant impact on drilling time or flexural strength. Nevertheless, drilling times did vary depending on the diameter of the drill. These findings provide valuable insights into the performance and durability of zirconia implant drills, contributing to the optimization of dental implant procedures.

## 1. Introduction

Dental implantation is now considered a standard procedure in dental practice. Single teeth, as well as multi-span restorations, can be anchored to dental implants using both fixed and removable methods. Success depends on many factors. Not only the dental implant but also the preparation of the implant bed represent significant factors for success [1].

The greatest influence on the survival of implants is exerted by the patient, clinician, and material.

Commonly used materials in implantology include titanium, titanium alloys, and medical steel. These materials have become the preferred choice for implant drills and dental implants in clinical practice [2]. Depending on the manufacturer, the composition of titanium may vary slightly. With the use of other materials, such as ceramics, attempts are being made to further improve critical aspects, such as lack of esthetics, alteration of homeostasis, and the oral microbiome, as well as the survival rate [3]. Dental ceramics are not all the same. Mainly, they are divided into three sub-types: silicate ceramics, oxide ceramics, and non-oxide ceramics [4]. Each type has different physical properties. A common representative of silicate ceramics is lithium disilicate, which is widely used in crown and bridge prosthetics [5]. Chosen for its translucency and sufficient flexural strength for single crowns and small bridges in the anterior region, the material has been the subject of critical discussion in the literature regarding its ability to meet mechanical demands as a supporting material in the posterior region [6]. If a so-called high-performance ceramic is required, the most well-known representative is zirconium dioxide, which is an oxide ceramic. Zirconium dioxide has up to four times the flexural strength of lithium disilicate. Because of its strong mechanical properties, zirconia is often used as a framework material in tooth- and implant-supported prosthetics [7]. One implant manufacturer is leveraging the mechanical properties of zirconia to improve its titanium implants [8]. However, zirconium implants are also offered by the industry, but there is less evidence supporting their use compared to titanium implants. With the introduction of zirconia implants, zirconia implant drills have also emerged on the market [9]. In a direct comparison of titanium and zirconia materials, noticeable differences in terms of thermal conductivity and brittleness are observed [10]. From a surgical perspective, it is crucial to critically consider the issue of heat generation during the preparation of implant beds using zirconia drills. Similarly, brittleness and edge stability also need to be considered. Titanium, like any metal, has a lower Young’s modulus than ceramics. This raises the question of whether implant drills made of zirconia can lead to abrasion and contamination of the implant bed with zirconia components, which could result in allergies [11].

From a clinical point of view, factors such as the surgeon’s experience, the length of the course, the duration of surgery, the timing of implant placement, the effectiveness of cleaning and sterilization of the products used, and the initial stability of the implant are all crucial for the long-term success of the implant. In addition to a short surgical time, the goal should be to minimize complications during the procedure. However, care must be taken to ensure that the heat generated during rotational preparation of the implant bed in the bone does not exceed 47 °C over one minute or 50 °C over 30 s to ensure optimal osseointegration [12,13]. Physically, the generation of frictional heat depends on several factors. These factors include bone density, water cooling, drill design and material, surgical time and the number of drills used, drilling speed, contact pressure, drill diameter, and implant drill sharpness [14].

The selection of materials and the design of the drill for implant drilling can influence the temperature [15]. The sharpness of the twist drill is an important factor in tissue-sparing osteotomies [16,17,18]. Heat generation can be significantly reduced if a sharp drill with low speed is used [19].

At this point, the function of the cardiovascular system and the associated heat exchange between the internal tissues of the body, organs, and skin are highlighted. The flow of blood transfers heat in the body and is, therefore, the most important method of heat exchange. This heat exchange can be influenced by exogenous and/or endogenous factors. Metabolic heat production increases during exercise [20].

The repeated use of drills and autoclave treatment affects drill sharpness and also impacts heat generation during drilling [21]. Studies by Ercoli et al. [16] and Elani et al. [22] indicated that the cutting efficiency of implant drills is reduced by repeated drilling and sterilization, which also increases the risk of thermal damage to bone tissue. Sterilization is a critical aspect of maintaining aseptic conditions and preventing the transmission of infectious agents in dental implant procedures. The sterilization process involves eliminating all forms of microbial life, including bacteria, viruses, and fungi, from the instruments and equipment used during implant surgery. The standard procedure for sterilization in dental practices often involves the use of autoclaves, which utilize high-pressure saturated steam to achieve sterilization. The instruments, including implant drills, are first cleaned to remove any debris or organic matter. Subsequently, they are placed in specially designed sterilization pouches or containers to maintain sterility throughout the process. The pouches or containers are then loaded into the autoclave, and the sterilization cycle is initiated. The autoclave subjects the instruments to high-pressure steam at a specific temperature and duration, effectively killing any microorganisms present. After the sterilization cycle is completed, the instruments are allowed to cool down before they can be safely handled and used in subsequent implant procedures. Proper sterilization procedures are essential for ensuring patient safety, minimizing the risk of postoperative infections, and maintaining the integrity of the implant surgery environment.

Therefore, this study aimed to analyze the influence of multiple uses of different implant drills on their cutting performance, as measured by drilling time and their bending strength. The hypothesis is that the use of drills and the number of sterilization cycles have no influence on either the drilling time or the flexural strength of zirconia implant drills.

## 2. Materials and Methods

**Figure 1:**
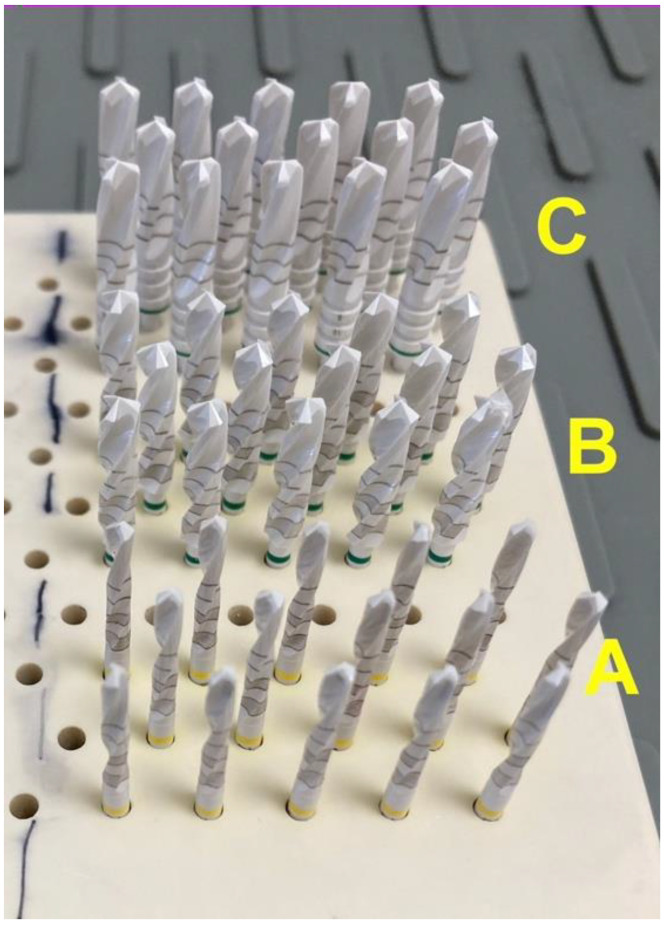
Zirconia implant drills from Z-Systems. **A**: implant drills with a 2.3 mm diameter, **B**: implant drills with a 3.75 mm diameter, **C**: implant drills with a 4.25 mm diameter.

The implant drills investigated were 60 twist drills manufactured by Z-Systems (Z-Systems AG, Oensingen, Switzerland). The drills are made of zirconium dioxide. They have a total working length of 16 mm and diameters of 2.3 mm (n = 20), 3.75 mm (n = 20), and 4.25 mm (n = 20) (Figure 1). The experiment was performed on fresh jaws from organic pigs. The porcine jaws were separated from the surrounding tissue and periosteum around the drilling area using a scalpel and raspatory. This was carried out to ensure that the chips were properly evacuated during the drilling process. Drilling was performed in the region of the angulus mandibulae (jaw angle) in the porcine jaw.

A custom-made examination apparatus was used for this experiment. This apparatus consists of two frames that are bolted together, with one frame positioned perpendicular to the other, edge to edge. The horizontal frame holds the pig’s jaw in position using screws. The vertical frame has a guide rail in the center, along which the test carriage can drill into the pig jaw with the drill in a perpendicular position. On the left and right sides of the frame, there are sensors that start and stop the time measurement using a light barrier. A weight can be attached to the extender of the carriage, to which the surgical contra-angle handpiece is connected, in order to simulate realistic contact pressure. The same surgical unit (W&H Elcomed 100 Surgical Console System; Nobel Biocare, Yorba Linda, CA, USA) was used for all osteotomies. The free-running speed of the drills was set to 800 rpm, and the maximum torque was set to 50 Ncm. The physiological solution (external irrigation) was automatically provided by the device, delivered to the drill, and maintained at a constant rate of 200 mL/min. A surgical contra-angle handpiece with a 20:1 ratio (WI-75 E/KM, W&H, Bürmoos, Austria) was attached to a guide rail.

In a preliminary test, the required contact pressure for the implant drills was determined. For this purpose, 11 dentists performed osteotomies in porcine jaws using a pilot drill. During drilling, the average contact pressure was determined with the aid of a pressure plate. The mean value of the contact pressure used was determined to be 20 N.

Before drilling, the pig’s jaw was immobilized, and the guide rail structure was adjusted until the drill made contact with the bone.

**Figure 2:**
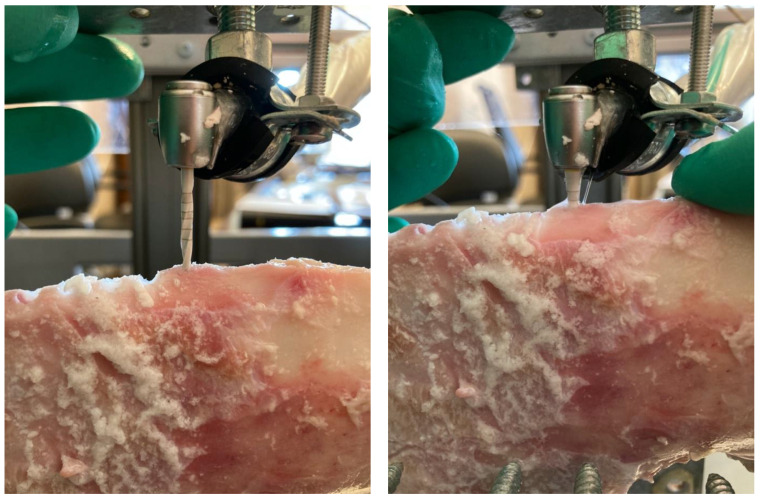
The (**left**) image shows the starting point of the measurement (where the drill tip touches the bone surface). The (**right**) image shows the end point of the measurement (the drilling depth of 13 mm has been reached).

The drilling time was determined by inductive proximity sensors. The sensors used had a detection frequency of 500 Hz (0.002 s). The sensors were placed at a depth of 13 mm, as per the desired drilling depth. The timing sensors were set so that recording began simultaneously with the start of drilling and ended after reaching a drilling depth of 13 mm (Figure 2). After each drilling session, all bone chips were removed from the drills using water.

The 60 drills were prepared for patient use in accordance with the manufacturer’s instructions and divided into four groups, with each group containing 15 drills (five drills of each type). The drills were applied in ascending diameter according to the manufacturer’s protocol.

**Figure 3:**
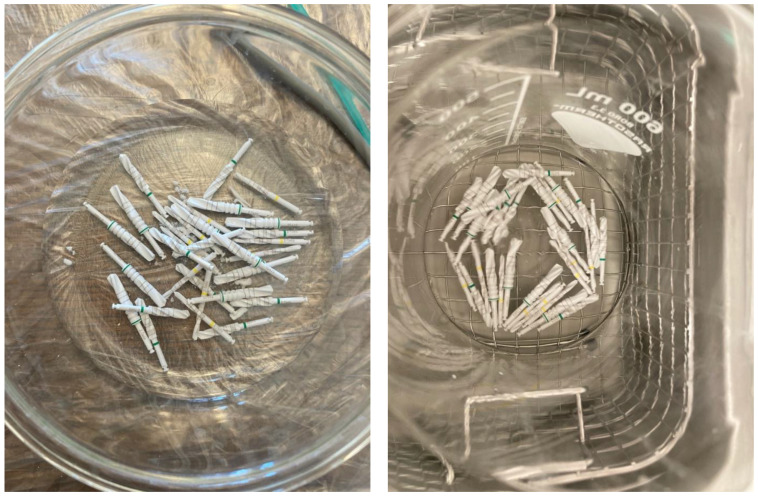

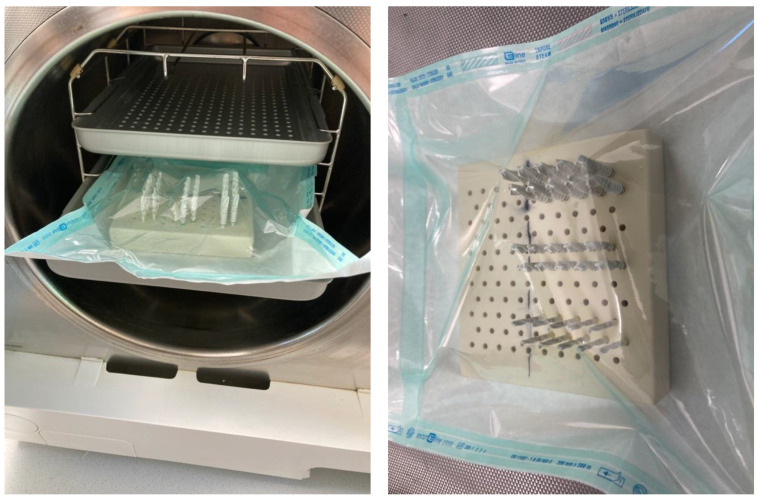
Instrument reprocessing sequence.

Successive drilling began with the ø2.3 mm drill, followed by the ø3.75 mm and ø4.25 mm drills. When the drill tunnel was reamed to a diameter of 4.25 mm, a new hole was drilled using a new trio of drills. The groups were assigned drilling cycle numbers. Group 1 was used once; Group 2 went through 10 drill cycles; Group 3 went through 20 drill cycles; and Group 4 went through 30 drill cycles. After each drilling cycle, the drills were cleaned and sterilized in accordance with the manufacturer’s instructions. According to the manufacturer’s manual, the reprocessing procedure included machine cleaning and disinfection in an ultrasonic bath (Elmasonic S 130H, Elma Schmidbauer GmbH, Singen, Germany) and sterilization in a vacuum autoclave (Universalprogramm, Vacuklav 40 B+, Melag, Berlin, Germany). During the experimental procedure, the drill bits were collected in a vessel filled with distilled water. Reprocessing began with mechanically cleaning the drills in an ultrasonic bath with a 2% disinfectant solution (ID 213 Instrument Disinfection, Dürr Dental SE, Bietigheim-Bissingen, Germany) for three minutes. This was followed by rinsing with distilled water and subsequent sterilization. The equipment was then packed in an autoclave (Figure 3). The results of the drilling time measurement were recorded in tabular form.

**Figure 4:**
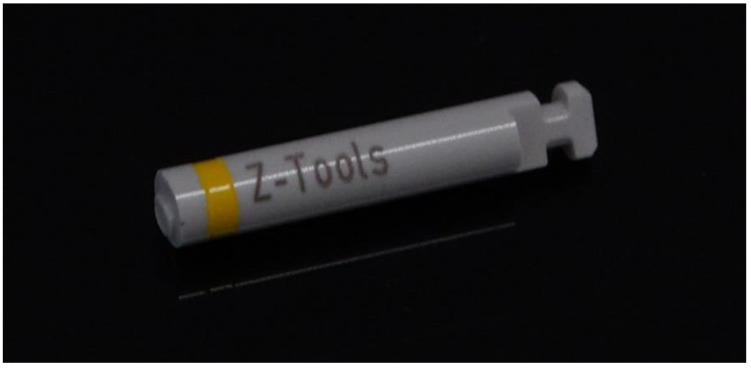
Cut-off drill shank.

**Figure 5:**
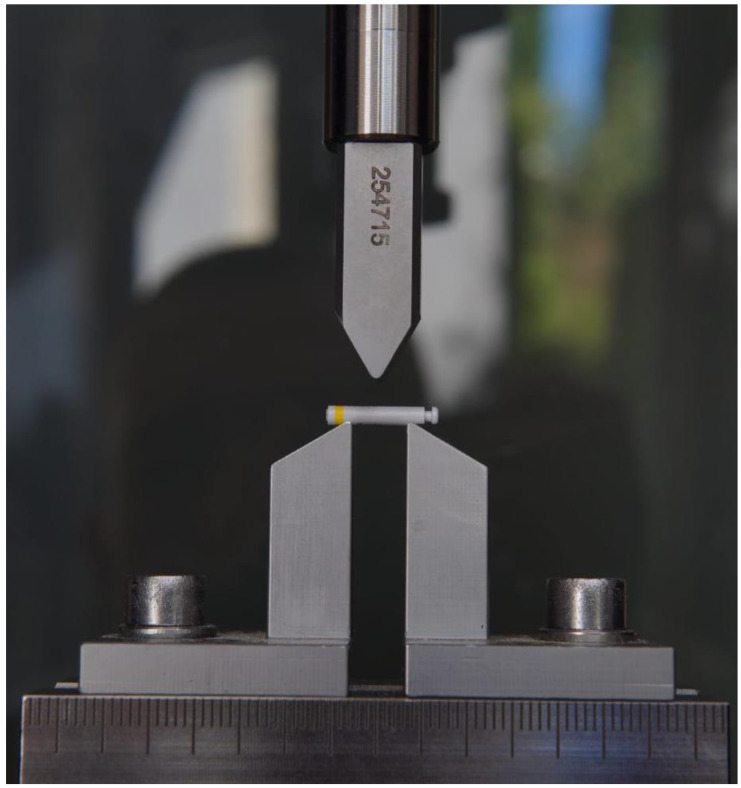
The 3-point bending test in the ZwickRoell testing machine.

**Figure 6:**
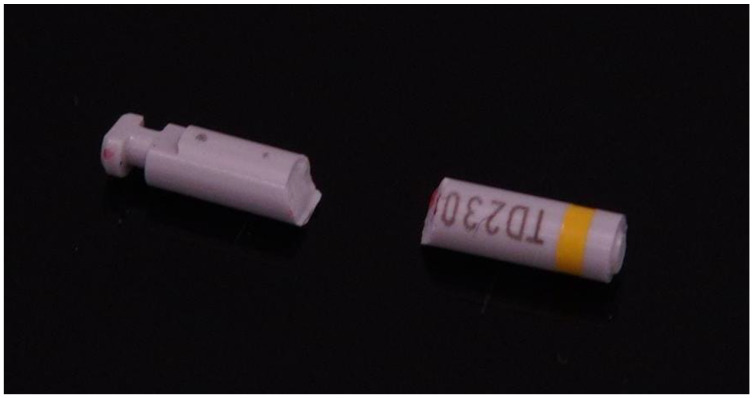
Broken drill shank after the 3-point bending test.

For the second investigation, the bending strength was tested by cutting through the drills using a rotating handpiece in the area between the drill portion and the drill shank after completing the first investigation (Figure 4). The shank was then used for the 3-point bending test. The cut drill shanks could then be individually secured in a universal testing machine (TNZ010, ZwickRoell, Ulm, Germany). The force measured during this process and the distance traveled were digitally recorded until breakage (Figure 5 and Figure 6). The data on maximum force and breaking force, measured in millimeters, were recorded and obtained through software (testing software testXpert III V1.4, ZwickRoell, Ulm, Germany).

Statistical analysis was performed using SigmaStat 4.0 (Systat Software GmbH, Düsseldorf, Germany). For statistical analysis, a parametric test called ANOVA analysis was performed. The significance level was set at 0.05.

## 3. Results

### 3.1. Drilling Time

The drilling times of all groups are normally distributed (*p* > 0.05). The results were analyzed using ANOVA tests.

#### 3.1.1. Repetitive Usage

**Figure 7:**
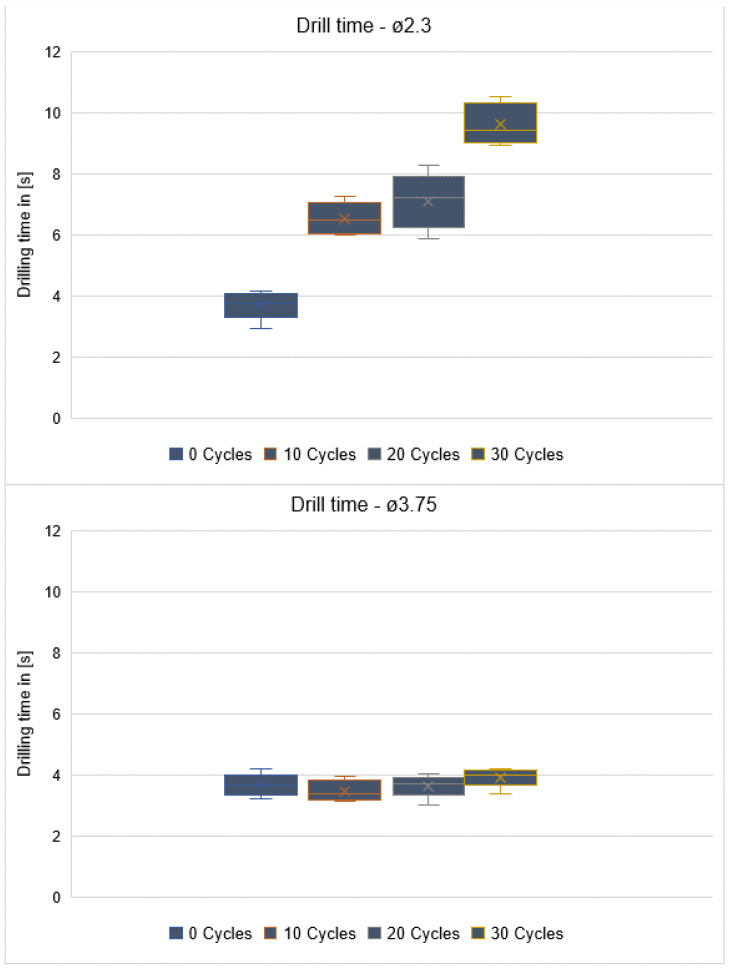

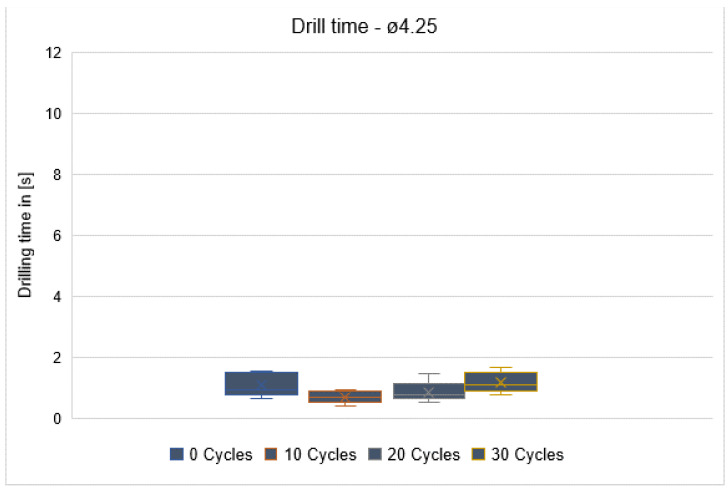
The three box-plot graphs (drill time: ø2.3, ø3.75, ø4.25) show the drilling time for each drill type after different stages of usage (after 0, 10, 20, and 30 cycles of usage).

A statistically significant difference was found in the pilot drills between the drilling times of groups 1 to 4 (*p* < 0.001). Mean values of 3.72 s ± 0.47 were found for Group 1, 6.54 s ± 0.53 for Group 2, 7.12 s ± 0.91 for Group 3, and 9.64 s ± 0.68 for Group 4. No statistically significant difference in drilling times was observed for the other drills: ø3.75 mm (*p* = 0.352) and ø4.25 mm (*p* = 0.137) (Figure 7).

#### 3.1.2. Diameter

Regardless of the number of times the drills were used, there were always statistically significant differences in drilling times between drills with diameters of 2.3 mm, 3.75 mm, and 4.25 mm (*p* < 0.001).

### 3.2. Flexural Strength

The drills with diameters of 2.3 mm and 4.25 mm were statistically normally distributed. The drills with a diameter of 3.75 mm did not exhibit a normal distribution of results. The normally distributed results were analyzed using a *t*-test. The non-normally distributed results were analyzed using the U-test.

**Figure 8:**
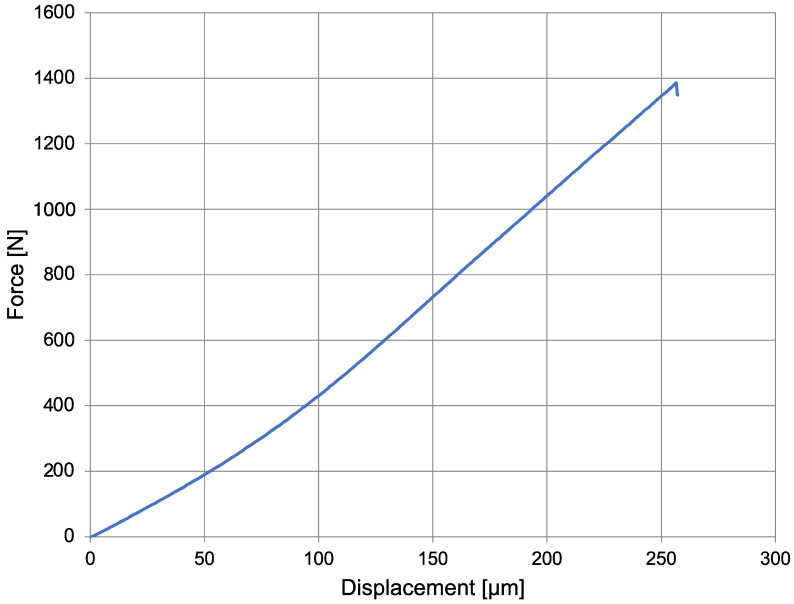
Force–displacement diagram: ø2.3 mm at 0 sterilization cycles.

As an example, a force–displacement diagram is shown here for the ø2.3 mm zirconia drill with zero sterilization cycles performed (Figure 8).

**Figure 9:**
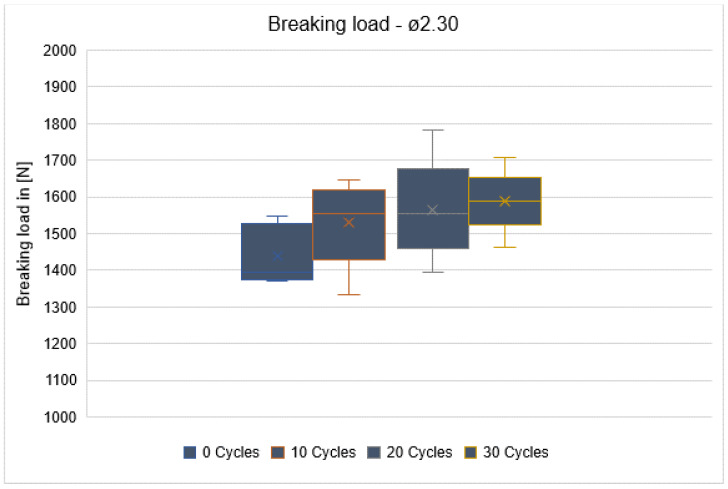

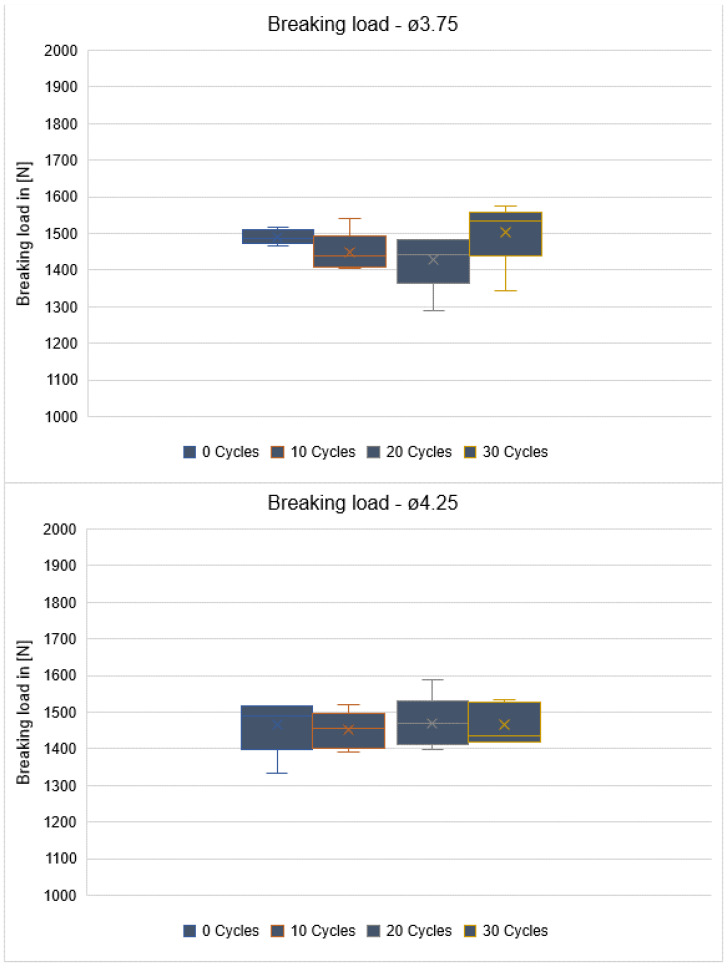
The three box plots (breaking load: ø2.3, ø3.75, ø4.25) show the breaking load for each drill type after different stages of usage (after 0, 10, 20, and 30 cycles of usage).

An almost linear increase in force was observed for all drill diameters and the number of sterilization cycles until the breaking load was reached. There were no statistically significant differences for all three diameters: ø2.3 mm (*p* = 0.185), ø3.75 mm (*p* = 0.425), and ø4.25 mm (*p* = 0.918) (Figure 9). This section was divided into subheadings. It should provide a concise and precise description of the experimental results, their interpretation, and the conclusions that can be drawn from the experiment.

## 4. Discussion

In this experimental study, various preparations of the implant site were performed in porcine jaws using zirconia implant drills of different diameters. Drilling times were measured, statistically evaluated, and subsequently compared. Additionally, the impact of drill usage and sterilization on their flexural strength was analyzed.

Reusable implant drills are typically made to withstand 10 surgical procedures. A study conducted by Medical Data International in the United States found that, on average, 2.5 implants are inserted per surgical procedure. This means that a reusable implant drill must maintain its surface finish and cutting performance for at least 25 tissue-preserving osteotomies [22]. Under study conditions, only the 2.3 mm diameter pilot drill exhibited a statistically significant increase in drilling time after 10 drilling and sterilization cycles (*p* < 0.01). In this study, the other drills showed no statistically significant differences. In a previous study investigating the influence of implant site preparation and sterilization on the performance and wear of implant drills made of medical steel, only the pilot drills showed a significant increase in drilling time after 30 drill cycles [23]. The findings of a study by Batista Mendes et al. [24] showed that zirconia drills exhibited smoother surfaces compared to stainless steel drills, which displayed more pronounced signs of wear. Koo et al. [25] concluded that it is recommended to replace the initial drill used in osteotomy preparation more frequently than the depth drills.

The material used was porcine mandibular bone. The porcine bone is described as a well-suited material for bone grafts because its cortical and cancellous structure is similar to that of humans and has comparable mechanical properties [19,26,27]. Regions of high (D1 and D2) and low (D3 and D4) density have been found in both porcine and human jaws [28]. Differences in bone quality, specifically the ratio of cortical to the cancellous bone in the drilling region, are a natural variation that occurs in both the porcine jaw and the human jaw. In the literature, several alternative models for simulating the human jaw are mentioned. There are commercially available synthetic jaw models specifically designed for dental drilling and implantation simulations. These models are made from materials that mimic the properties of human bone and soft tissue [29]. Though not as readily accessible as porcine mandibles, cadaveric specimens can be obtained for research purposes. They provide a closer approximation to human anatomy and can be used for drilling trials [30]. With the advancement of 3D printing technology, it is now possible to create customized jaw models using patient-specific data. 3D printed models can replicate the complexity of the human jaw and offer a cost-effective alternative for drilling trials [31]. Apart from porcine mandibles, other animal jaw models can be used, such as bovine or sheep mandibles. These can provide a similar structural and anatomical representation of the human jaw [32]. Regarding synthetic jaw models, several differences in drill behavior arise in comparison to porcine mandibles.

Synthetic jaw models, specifically designed for dental simulations, may exhibit slight variations in mechanical properties compared to porcine mandibles. These differences can affect drilling behavior, including drill penetration, resistance, and heat generation. However, they still offer a viable alternative for simulating dental procedures [33].

The axial drilling pressure was kept constant at 20 N, as determined in a preliminary test involving 11 dentists. In the literature, the axial pressure exerted on the surgical contra-angle handpiece is recognized as a factor that affects heat generation. However, statements regarding the appropriate value vary. Values range from nonspecific recommendations of low contact pressure to values of 12 N to contact pressures of 20 N or 24 N [17,34,35,36].

Drilling was performed using both the pilot drill and the extension drill at a speed of 800 rpm. This speed is in accordance with the manufacturer’s recommendation and is also within the range of rotations per minute postulated by many authors [12,37,38,39,40].

Regarding the effect of autoclave treatment on cutting efficiency, a 1:1 ratio was determined for the relationship between implant bed preparation and sterilization cycles. Even though multiple implants are often inserted during each surgical procedure, this ratio is still relevant for daily practice. 

In this study, the cutting performance of the implant drills was evaluated based on the time it took to reach a drilling depth of 13 mm while maintaining constant pressure. While the pilot drills showed a statistically significant increase in drilling time, the other drills showed no significant difference in drilling time. For the pilot drills, the significant impact of drilling cycles on cutting performance can be attributed to the need to penetrate the intact cortical bone, resulting in increased stress in the apical drill area. In contrast, the depth drills had no cortical bone to penetrate.

In addition, the drill geometry has an influence on the cutting performance [17,41]. All drills are twist drills. The pilot drills have only two cutting edges, whereas the extension drills have three. A study by Watanabe et al. [27] showed an increase in cutting efficiency when the number of cutting edges was increased. From a practical standpoint, in order to maintain the recommended rotation speed of 800 rpm, the only option would be to increase the axial pressure load to compensate for the geometric disadvantage. Increasing this parameter could reduce the time required for preparation or allow better penetration into the cortical bone initially, but it would also lead to an increase in temperature [34].

The higher the load on the drill, the higher the frictional heat [42]. The heat generation and wear of the drill can be influenced by varying the contact pressure. The influence of heat development on bone has already been demonstrated in studies [34]. While measuring the temperature at the drill head, a constant force was applied to the surgical handpiece. As a result of the applied pressure, the temperature of the bone can increase even more due to its density. The much denser and firmer cortical bone builds up considerably more counterpressure than the adjacent cancellous bone. However, the literature has not yet described the increased wear on the implant drill caused by thicker or thinner compacta. 

Every cleaning and sterilization process subjects every drill to thermal, pressure-related, and mechanical stress. This might lead to wear effects on the cutting flutes [23]. Because of the low risk of infection and the excessively high material costs associated with single-use, it is generally standard practice to sterilize the implant drills [43,44,45]. 

The zirconia used in the test exhibits good mechanical properties as well as sterilizability [25]. With the three-point bending test, an average force of 1484.89 N was achieved to cause a fracture. This is consistent with the study conducted by Maccauro et al. [46] who discovered that zirconia material has interesting properties from both a mechanical and biocompatibility perspective, making it suitable for dental devices. 

As a potential alternative to steel, zirconium could broaden the market for implant drills. Its material properties and the findings of the present study support this notion. Currently, zirconia drills are manufactured by milling, which is expensive [47]. Other types of ceramics, such as lithium disilicate, can be shaped through injection molding in addition to milling, resulting in significant cost reductions. Companies are working on the 3D printing of ceramic materials [48]. Lithoz GmbH, based in Vienna, offers freely designable 3D-printed parts made of yttrium-reinforced zirconia. LithaCon 3Y 210 (Lithoz GmbH, Vienna, Austria) contains zirconia stabilized with 3 mol% yttria. With the reduction of manufacturing costs, we can expect a broader range of materials to become available on the market. The possibility of material abrasion and contamination in the operating area must be examined.

## 5. Conclusions

Within the limitations of the present study, several noteworthy observations emerge, shedding light on the performance and wear of implant drills:

Firstly, the flexural strength of the implant drills remains consistent, implying their mechanical integrity is not compromised during the testing process.

Secondly, the geometry of the implant drill has a dual impact on cutting efficiency and durability, suggesting that certain designs may yield better results in these aspects.

Thirdly, extension drills exhibit a non-significant change in drilling times, suggesting they can be used multiple times without significant loss of cutting performance.

Fourthly, pilot drills experience a significant change in drilling times, suggesting wear and potential degradation of their cutting ability over time.

Lastly, the sterilization process could be a crucial factor affecting the wear of the main cutting edges of the drills.

To gain a more comprehensive understanding of how implant bearing preparation and sterilization precisely influence the performance and wear of implant drills, further research is warranted. To delve deeper into the mechanisms underlying the observed changes in implant drills’ performance and wear, the next investigation should involve scanning electron microscope (SEM) examination. SEM can provide high-resolution images, allowing for a detailed analysis of the surface morphology and wear patterns of the drill bits.

Additional investigations can help refine the current findings and provide valuable insights for dental professionals seeking to optimize their implant procedures. Expanding the scope of the research can lead to more effective and reliable dental implant treatments in the future. The pursuit of such knowledge is vital for improving patient outcomes and enhancing dental care practices.

## Data Availability

Data are contained within the article.
